# Swarming motility and biofilm formation of *Paenibacillus larvae*, the etiological agent of American Foulbrood of honey bees (*Apis mellifera*)

**DOI:** 10.1038/s41598-018-27193-8

**Published:** 2018-06-11

**Authors:** Anne Fünfhaus, Josefine Göbel, Julia Ebeling, Henriette Knispel, Eva Garcia-Gonzalez, Elke Genersch

**Affiliations:** 1 Institute for Bee Research, Department of Molecular Microbiology and Bee Diseases, Hohen Neuendorf, Germany; 2 Freie Universität Berlin, Fachbereich Veterinärmedizin, Institut für Mikrobiologie und Tierseuchen, Berlin, Germany

## Abstract

American Foulbrood is a worldwide distributed, fatal disease of the brood of the Western honey bee (*Apis mellifera*). The causative agent of this fatal brood disease is the Gram-positive, spore-forming bacterium *Paenibacillus larvae*, which can be classified into four different genotypes (ERIC I-IV), with ERIC I and II being the ones isolated from contemporary AFB outbreaks. *P. larvae* is a peritrichously flagellated bacterium and, hence, we hypothesized that *P. larvae* is capable of coordinated and cooperative multicellular behaviors like swarming motility and biofilm formation. In order to analyze these behaviors of *P. larvae*, we firstly established appropriate functional assays. Using these assays we demonstrated that *P. larvae* ERIC II, but not *P. larvae* ERIC I, was capable of swarming. Swarming motility was hampered in a *P. larvae* ERIC II-mutant lacking production of paenilarvin, an iturin-like lipopeptide exclusively expressed by this genotype. Both genotypes were able to form free floating biofilm aggregates loosely attached to the walls of the culture wells. Visualizing the biofilms by Congo red and thioflavin S staining suggested structural differences between the biofilms formed. Biofilm formation was shown to be independent from paenilarvin production because the paenilarvin deficient mutant was comparably able to form a biofilm.

## Introduction

The Western honey bee *Apis mellifera* is a generalist pollinator and managed colonies of *A. mellifera* are widely used in global agriculture for the pollination of many crops and fruit grown in the open field^1,2^. In addition, *A. mellifera* also pollinates numerous wild flowers thus contributing to biodiversity in natural ecosystems. Thus, infectious diseases threatening the performance and survival of individual honey bees and honey bee colonies^3^ are of great concern not only for farmers and beekeepers but also for the general public. Among the pathogens posing the most serious threats to honey bees is *Paenibacillus larvae*. This Gram-positive, spore-forming bacterium is the causative agent of American Foulbrood (AFB) of honey bees^4^. *P. larvae* only infects bee larvae, hence, AFB only affects the bee brood but still the disease is able to kill entire colonies if left untreated^5^. *P. larvae* and AFB are globally distributed in *A. mellifera* populations. AFB is highly contagious and it may spread quite fast within and between honey bee colonies and apiaries. In most countries, it is classified as notifiable disease; control measures are regulated by corresponding laws and often include culling of diseased colonies.

The species *P. larvae* comprises the four genotypes ERIC I–IV which have been defined by repetitive element PCR (repPCR) performed with primers amplifying enterobacterial repetitive intergenic consensus elements (ERIC primers)^4^. However, other methods like MALDI-ToF analysis^6^, multi locus sequence type (MLST)^7^ or multiple locus variable number of tandem repeat analysis (MLVA)^8^ are also able to differentiate these four genotypes. This genotype differentiation is practically relevant because the four genotypes differ in several phenotypic features^4,9^ including virulence at both the level of the individual larva and the colony^10,11^.

Contemporary outbreaks of AFB all over the world are caused by two of the four genotypes only, by *P. larvae* ERIC I and ERIC II^7^. Therefore, these two genotypes have received much attention in recent research. Genome analysis of a non-genotyped strain of *P. larvae* provided first indications of possible virulence factors^12^, but it was the comparative genome^13,14^ and proteome analysis^15^ that yielded promising candidates, which could then be confirmed experimentally as virulence factors. On the basis of the previous and newly gained knowledge on *P. larvae*, the following picture emerges of the pathogenesis of *P. larvae* infections: In individual larvae, the infection process starts with the oral uptake of *P. larvae* spores during the first 36 hours after egg hatching. After ingestion, the spores germinate in the midgut lumen. The vegetative bacteria proliferate massively until they fill the entire gut lumen^16^. Eventually, the bacteria start to attack the midgut epithelium with the help of various virulence factors. The chitin-degrading enzyme *Pl*CBP49 is a key virulence factor for both genotypes and is responsible for the degradation of the peritrophic matrix that is supposed to protect the epithelium from attack by pathogens^17,18^. The surface layer protein SplA is specifically expressed by *P. larvae* ERIC II and mediates bacterial adhesion to the midgut epithelium, a step that is obviously relevant for the pathogenic strategy of *P. larvae* ERIC II^15,19^. The two toxins Plx1 and Plx2 exclusively expressed by *P. larvae* ERIC I were shown to be relevant for the virulence of this genotype^20,21^. For the recently described toxin C3larvin^22^ encoded by the loci Tx7 and TxIII in the genomes of *P. larvae* ERIC I and ERIC II^13^, respectively, it has yet to be shown experimentally that it is a virulence factor. The aforementioned virulence factors are involved when *P. larvae* attacks and breaches the midgut epithelium and invades the hemocoel thereby killing the larva. Subsequently, the larval cadaver is degraded by *P. larvae* to a ropy mass which dries out to a scale. While the ropy mass can still be removed from the brood cell, the scale tightly adheres to the cell wall and resists the cleaning attempts of the bees.

When searching for virulence factors, the non-ribosomal peptides (NRP) and peptide-polyketide (NRP/PK) hybrids of *P. larvae*, the biosynthetic machineries of which are encoded by complex giant gene clusters^23^, attracted special attention. The novel tripeptide sevadicin produced by *P. larvae* ERIC II has antibacterial activity^24^. Another novel NRP/PK hybrid molecule is paenilamicin, produced by *P. larvae* ERIC I and ERIC II, which is active against some bacteria and fungi^25^ and plays a role in outcompeting microbial competitors of *P. larvae* in the larval midgut^26^. Bacillibactin, a catechol-type siderophore known from members of the *Bacillus cereus sensu lato* group and *B. subtilis*, is synthetized by both *P. larvae* genotypes only under iron depletion conditions^27^. In addition, *P. larvae* ERIC II non-ribosomally produces a group of lipopeptides belonging to the iturin-family, the so-called paenilarvins^28^. Paenilarvins have strong antifungal activity^28^ but lack cytotoxicity^29^. Hence, although a direct role as virulence factor during the invasive phase of pathogenesis could not be established for the paenilarvins^29^, they might be involved in outcompeting fungal competitors of *P. larvae* during the bacterial lifecycle in infected larvae.

*P. larvae* has been described as peritrichously flagellated and highly motile^4^ and, indeed, genes for building flagella were found in its genome^12^ suggesting that *P. larvae* is capable of coordinated activity like swarming motility and biofilm formation. Swarming motility is defined as a coordinated, flagella-driven movement of a collective group of bacteria across a surface^30^. A variety of Gram-negative and Gram-positive bacterial species exhibit swarming motility^31^. Within the genus *Paenibacillus*, several flagellated species like *P. alvei*^32^, *P. dendritiformis*^33^, and *P. vortex*^34^ show swarming behaviour. Swarming is generally thought to facilitate rapid colonization of nutrient-rich environments and to accelerate biomass production^31^. Peritrichously arranged flagella and the tightly controlled production of an extracellular matrix consisting of polysaccharides, biosurfactants, peptides, and proteins are required in many cases for swarming motility to occur^35^.

Many bacterial species are able to switch between planktonic growth and biofilm formation. The broadest definition of a biofilm is that it represents cooperatively acting microorganisms forming cell clusters which are held together and protected from adverse external influence by an extracellular matrix secreted by the members of the biofilm. This matrix is a characteristic hallmark of bacterial biofilms and consists of exopolysaccharides, secreted proteins, amyloid fibres, and sometimes nucleic acids^36,37^ (and references therein). Sessile biofilms are found attached to both biotic or abiotic surfaces^38,39^. However, bacteria can also form floating biofilms (‘pellicles’) at air-liquid interfaces^38^ or even form free floating biofilms^40^. For the formation of sessile biofilms, different developmental stages are described starting from the initially reversible, later then irreversible surface attachment of planktonic cells followed by the formation of microcolonies until the final macrocolonies are formed. From these macrocolonies, cells are dispersed to become planktonic again or the entire macrocolony can detach or dissolve^39^. Biofilms are of tremendous medical relevance because they not only protect bacteria from the host’s immune system but also render them refractory against antimicrobial treatment^41–44^. Not surprisingly though, that biofilms are considered the preferred lifestyle of bacteria when living in a host or on host tissues^45^.

Swarming and biofilm formation might be important during various stages of the lifecycle of *P. larvae* in infected larvae. It was recently speculated that exopolymeric substances (EPS) as structural components of the extracellular matrix during biofilm development could possibly be involved in virulence and tolerance to physiological stress in *P. larvae*^46^. However, no data actually proving biofilm formation or substantiating the existence and role of *P. larvae* biofilms in virulence was presented. Therefore, neither swarming nor biofilm formation have been demonstrated for this bacterial species so far. We here present our data showing that *P. larvae* ERIC II but not *P. larvae* ERIC I is capable of swarming motility. We provide evidence that paenilarvin, the recently identified iturin-like secondary metabolite of *P. larvae* ERIC II^28,29^, is involved in swarming motility. We also demonstrate that both genotypes indeed form biofilms but that their formation is not influenced by paenilarvin. These assays for *P. larvae* swarming motility and biofilm formation address two hitherto unknown characteristics of this bacterium which might be relevant for *P. larvae* when it is colonizing the infected or degrading the dead larva. Hence, understanding *P. larvae* swarming motility and biofilm formation might contribute to a better understanding of the disease processes and pave the way for novel treatment strategies.

## Results

### Swarming motility of *P. larvae*

**Figure 1:**
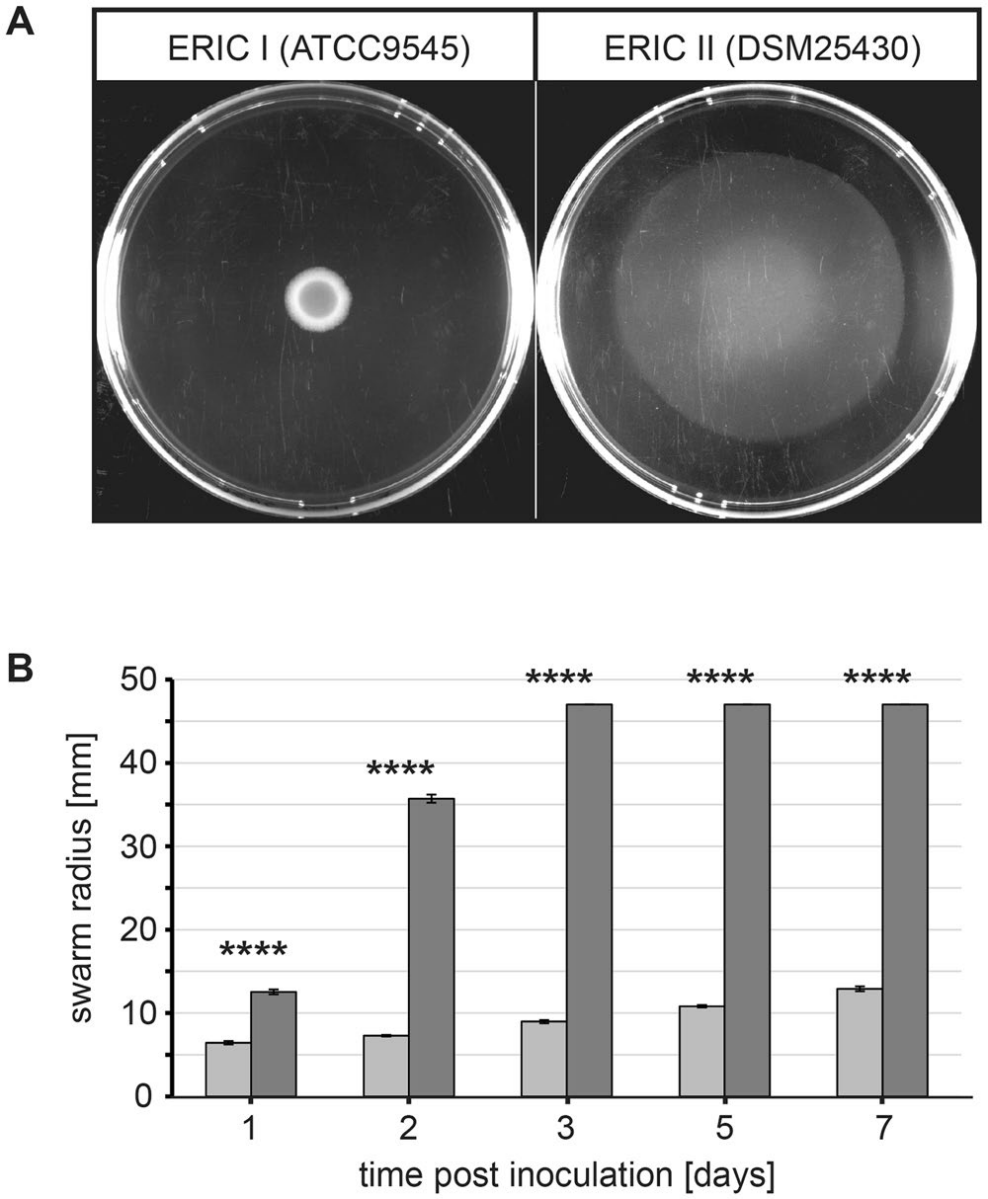
Swarming motility of *P. larvae* ERIC I (ATCC9545) and *P. larvae* ERIC II (DSM25430). Bacterial suspensions (10 µl) with an OD_600_ of 0.1 were inoculated centrally onto brain heart infusion (BHI; 0.5%) agar plates and incubated for two days under aerobic conditions at 37 °C. For both genotypes, three biological replicates with three technical replicates each were performed. (**A**) A representative plate is shown for each strain. Uncolonized agar is black and bacterial biomass is milky white to white. (**B**) Swarming motility was monitored over time and swarm radii were measured for each strain at one, two, three, five, and seven days post inoculation. Three biological replicates with three technical replicates each were performed. Values are given as mean ± SEM. Statistical analysis of the difference in swarming motility between ATCC9545 (light gray columns) and DSM25430 (dark gray columns) was performed with an unpaired Student’s t-test for each time point (not significantly different: n.s., p ≥ 0.05; significantly different: *p < 0.05; **p < 0.01; ***p < 0.001; ****p < 0.0001).

**Figure 2:**
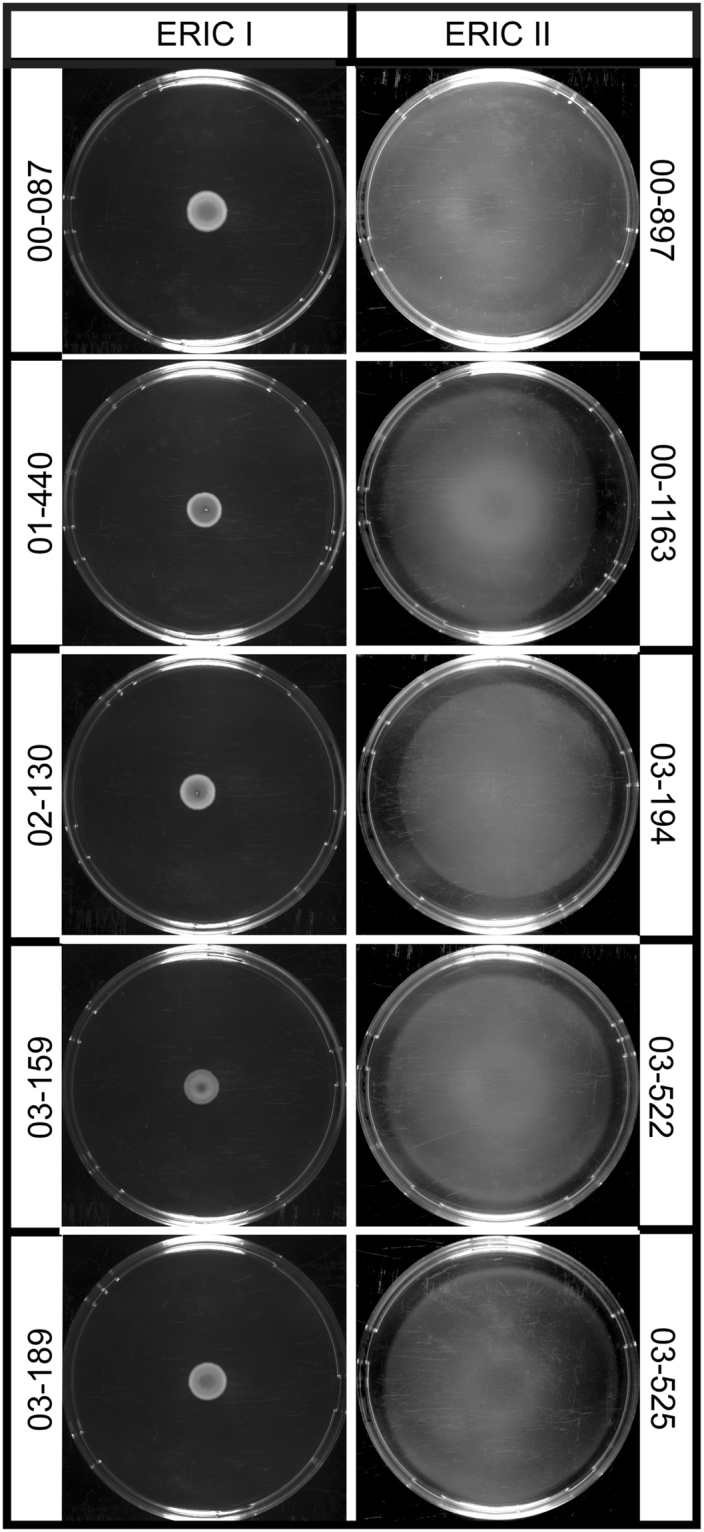
Swarming motility of field isolates of *P. larvae* ERIC I and *P. larvae* ERIC II. Swarming assays were performed with five strains each of *P. larvae* ERIC I and ERIC II which had been isolated from contemporary AFB outbreaks (field isolates). For all strains, three biological replicates with three technical replicates each were performed and a representative plate is shown for each strain. Uncolonized agar is black and bacterial biomass is milky white to white. Swarming was only evident for *P. larvae* ERIC II strains.

To test the hypothesis that *P. larvae* is able to perform a multicellular flagellum-driven surface movement known as swarming motility, we first established an appropriate experimental procedure based on soft agar plates (0.5%) made from brain heart infusion broth (BHI) and on inoculating 10 µl of a bacterial suspension with an OD_600_ of 0.1 centrally onto the agar plate. While *P. larvae* ERIC I did not show swarming motility (Fig. 1A), *P. larvae* ERIC II reproducibly exhibited swarming motility under these conditions (Fig. 1A). Time course experiments performed by measuring the swarm radii at different time points (Fig. 1B) revealed that *P. larvae* ERIC II was able to cover the entire surface of the plate within three days (swarm radius 47 mm). In contrast, the macrocolonies formed by *P. larvae* ERIC I had reached a size of only 12.91 mm ± 0.29 mm (mean ± SEM) even after seven days. The radii of the area covered by *P. larvae* ERIC II were significantly different from those covered *P. larvae* ERIC I for all time points (p < 0.0001, unpaired Student’s t-test). Next, we randomly selected another five field strains of each *P. larvae* genotype and tested their swarming behaviour. All *P. larvae* ERIC I strains formed macrocolonies but were not able to swarm while all tested *P. larvae* ERIC II strains nearly covered the entire surfaces of the plates within two days (Fig. 2). These results suggested that swarming behaviour is another feature distinguishing these two genotypes.

### Biofilm formation of *P. larvae*

**Figure 3:**
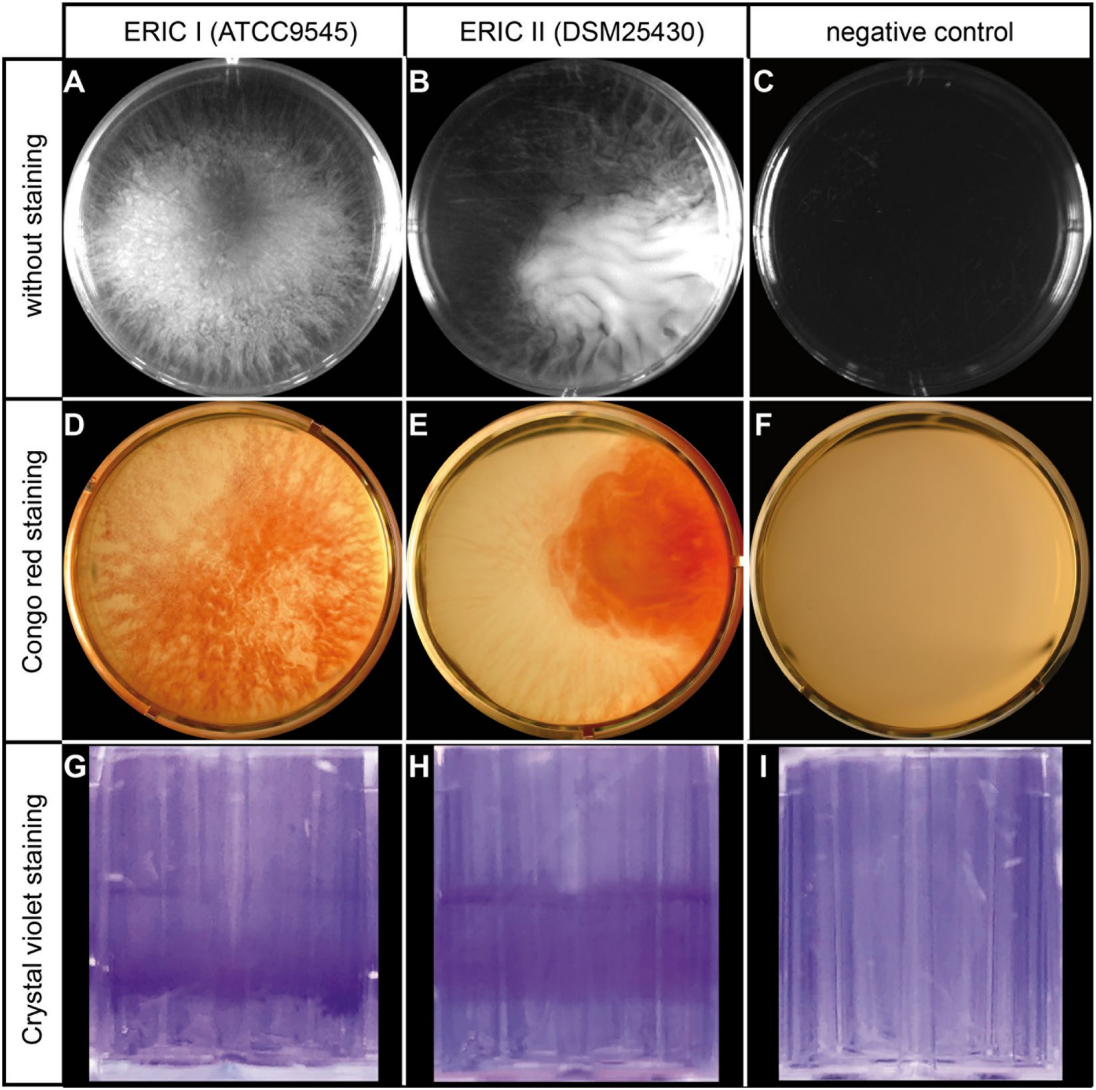
Biofilm formation of *P. larvae* ERIC I (ATCC9545) and *P. larvae* ERIC II (DSM25430) cultivated in static liquid. Bacterial suspensions of *P. larvae* ERIC I (ATCC9545; **A**,**D**,**G**) and *P. larvae* ERIC II (DSM25430; **B**,**E**,**H**) in brain heart infusion (BHI) broth were incubated without agitation in six-well-plates at 37 °C for five (**A**,**B**) and six (**D**,**E**) days, or in 96-well-plates for six days (**G**,**H**). For each *P. larvae* genotype, three biological replicates were performed both for obtaining pictures under unstained conditions (**A**,**B**), after Congo red staining of the extracellular matrix (**D**,**E**), and Crystal violet staining of the bacterial cells adherent to the well walls (**G**,**H**). Negative controls for each assay are shown in (**C**,**F**, and **I**). Representative pictures are shown.

To test the hypothesis that *P. larvae* is capable of forming multicellular aggregates known as biofilms we established culture conditions that resulted in the formation of free floating bacterial aggregates that were visible to the naked eye and resembled biofilms (Fig. 3A,B). These aggregates reproducibly formed after five to six days when bacterial suspensions were incubated without agitation (static culture) at 37 °C whereas no such aggregates were visible in the negative controls (Fig. 3C). While the aggregates formed by *P. larvae* ERIC I appeared “hairy” with protrusions attaching all around to the wall of the well (Fig. 3A), the *P. larvae* ERIC II aggregates had a rather laminar appearance (Fig. 3B).

A characteristic hallmark of biofilms is that the bacteria are surrounded by an extracellular matrix which contains i.a. exopolysaccharides and amyloid fibers^36,37^. Hence, dyes specifically staining these extracellular matrix components can be used to specifically visualize biofilms^47–49^. To determine whether the observed *P. larvae* aggregates (Fig. 3A,B) indeed represented biofilms, that is that the bacteria were surrounded by an extracellular matrix, we used the amyloidophilic dyes Congo red and thioflavin S. Both dyes stain polysaccharides and amyloid fibers^50–53^ present in the extracellular matrix produced by bacteria during biofilm formation and for stabilizing the biofilm^48,54–58^ and, therefore, are routinely used to test for bacterial biofilm formation.

Upon Congo red staining, the multicellular aggregates (Fig. 3A,B) stained red while the surrounding medium remained unstained (Fig. 3D,E) like the negative control which represented CR-stained medium (Fig. 3F). These results indicated that both *P. larvae* ERIC I and ERIC II aggregates were embedded in extracellular matrix and, hence, that the bacteria formed biofilms under the chosen conditions. The biofilms though floating again appeared to have contact to the walls of the wells. Consequently, bacterial cells adherent to the well walls could be visualized by Crystal violet staining (Fig. 3G,H). No staining was evident in the negative controls (Fig. 3I).

**Figure 4:**
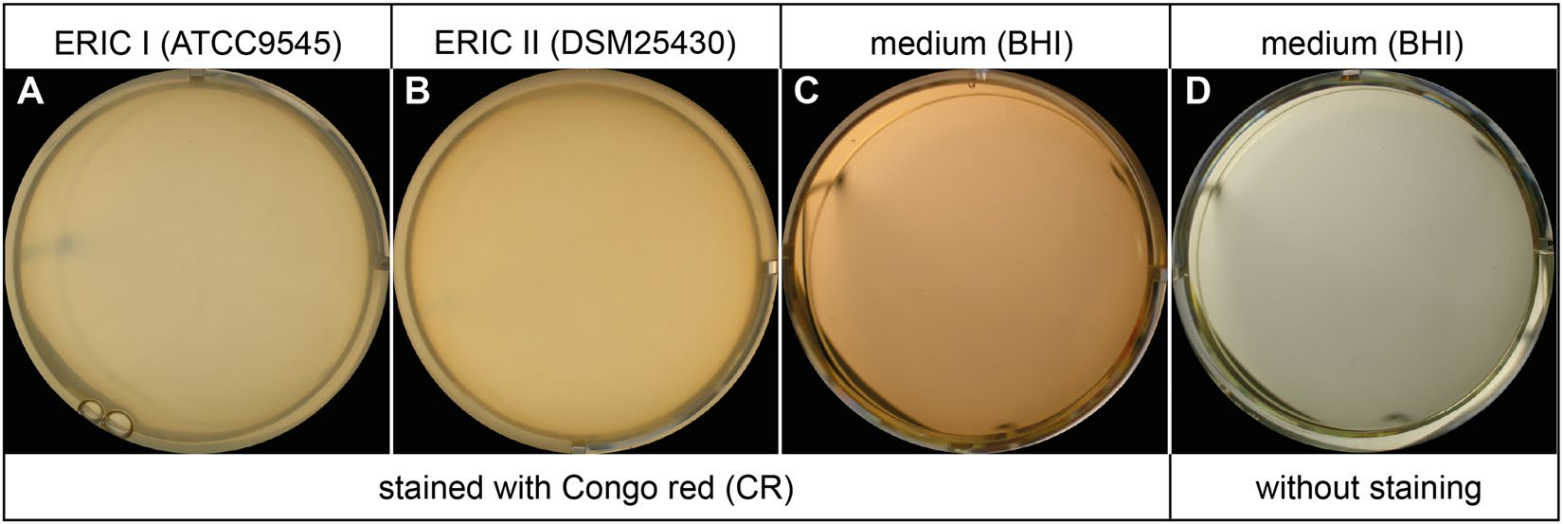
Planktonic cells of *P. larvae* ERIC I (ATCC9545) and *P. larvae* ERIC II (DSM25430). Bacterial suspensions of *P. larvae* ERIC I (ATCC9545; **A**) and *P. larvae* ERIC II (DSM25430; **B**) in brain heart infusion (BHI) broth were incubated under constant agitation to prevent biofilm formation. Cultures of planktonic cells were stained with Congo red (**A**,**B**) while BHI medium stained with Congo red (**C**) or without staining (**D**) served as negative controls. Representative pictures are shown.

In order to further substantiate that CR specifically stained the extracellular matrix surrounding *P. larvae* cells organized in a biofilm, we next tried to stain planktonic *P. larvae* cells, which were obtained by culturing *P. larvae* under constant agitation thereby preventing biofilm formation. Incubating such a non-static culture of *P. larvae*, hence, of planktonic *P. larvae* cells, with CR (Fig. 4A,B) did not reveal any stained aggregates (for comparison see Fig. 3D,E) but instead the wells looked like the negative controls (Figs 3F, 4C,D). These results suggested that the aggregates which formed when *P. larvae* was cultivated in static liquid and which were visualized by using the biofilm specific dye CR (Fig. 3A–F) were indeed *P. larvae* biofilms.

**Figure 5:**
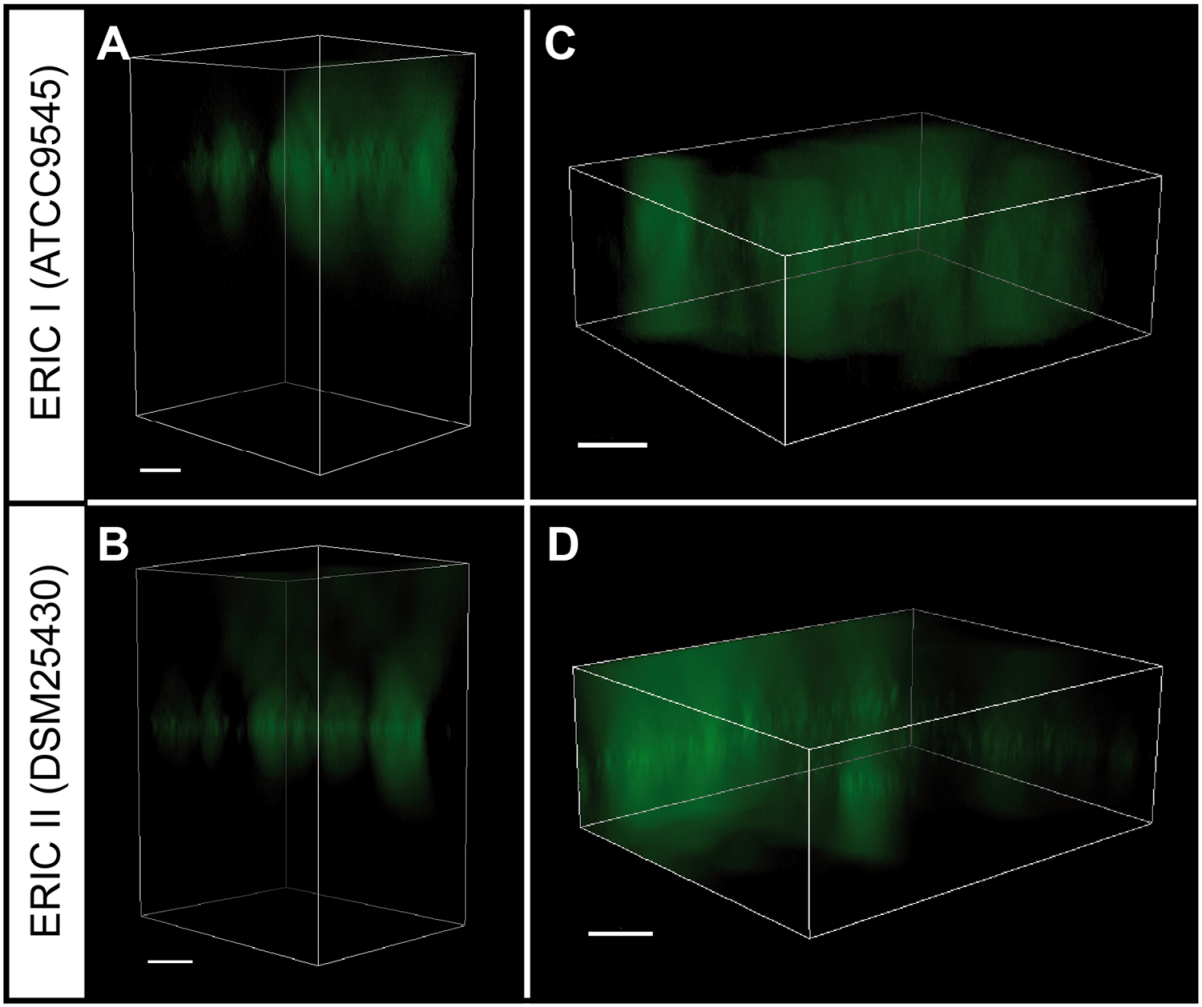
Fluorescence microscopy of *P. larvae* floating biofilms. Bacterial suspensions of *P. larvae* ERIC I (ATCC9545; **A**,**C**) and *P. larvae* ERIC II (DSM25430; **B**,**D**) in Sf-900 II SFM medium supplemented with 30 µg/ml thioflavin S were incubated without agitation in 96-well-plates at 37 °C for six days. The thioflavin S-stained extracellular matrix in the floating biofilms was visualized using fluorescence microscopy; Z-stack processing was performed to obtain three-dimensional images of the wells containing the biofilms (**A**,**B**) and the region within the wells where the biofilms were located (**C**,**D**). Bars represent 20 µm.

In order to provide further evidence for *P. larvae* biofilm formation, we next examined the three-dimensional structures of the bacterial aggregates by thioflavin S staining and semi-confocal fluorescence microscopy allowing to generate Z-stacks by incrementally stepping through the samples. Green fluorescence indicative for the presence of amyloid fibers was observed in a defined structured zone of the well as floating mirror-image ellipsoidal clouds each having a more densely fluorescing “axis of reflection” (Fig. 5A,B) which seemed to be more pronounced in DSM25430 than in ATCC9545 (Fig. 5C,D). The *P. larvae* biofilms neither adhered at the bottom of the well nor formed at the liquid-air interface (pellicle) but were rather levitating in the well (Fig. 5) like free floating biofilm aggregates recently described for *Pseudomonas aeruginosa*^40^.

### Influence of paenilarvin on swarming motility and biofilm formation

Paenilarvins are iturin-like secondary metabolite specifically produced by *P. larvae* ERIC II^28^. Because iturin-like secondary metabolites are known to influence the motility of their bacterial producers^59,60^, we tested whether paenilarvin has an influence on swarming motility or biofilm formation of *P. larvae* ERIC II. We used the newly established assays for swarming motility and biofilm formation and compared the behaviour of wild-type *P. larvae* DSM25430 and of an inactivation mutant for the paenilarvin gene cluster, *P. larvae* DSM25430 Δ*itu*, which is deficient in paenilarvin production^29^.

**Figure 6:**
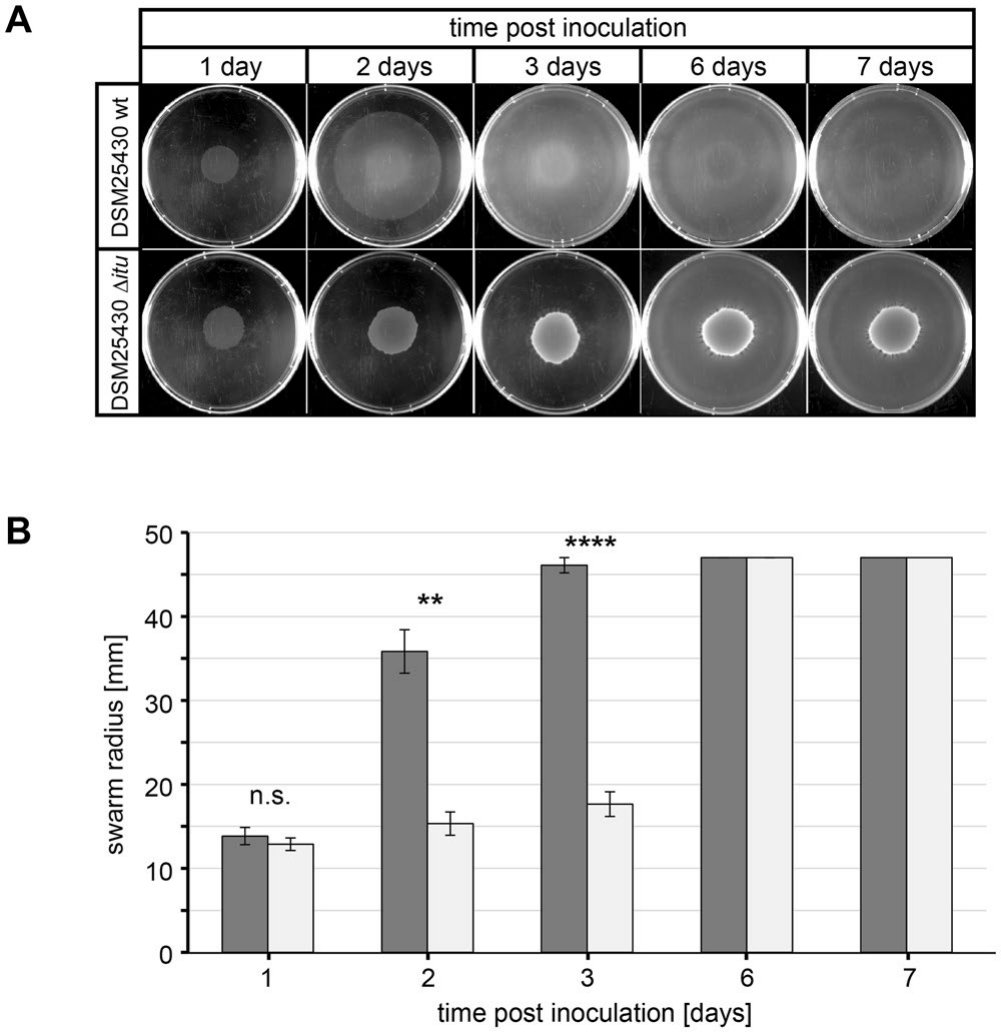
Involvement of paenilarvin in swarming motility of *P. larvae* ERIC II. Wild-type *P. larvae* ERIC II (DSM25430 wt) and a corresponding inactivation mutant for the paenilarvin gene cluster (DSM25430 Δ*itu*) were tested in swarming assays. Bacterial suspensions (10 µl) with an OD_600_ of 0.1 were inoculated centrally onto a brain heart infusion (BHI; 0.5%) agar plate and incubated at 37 °C for seven days. Pictures were taken at one, two, three, six, and seven days post inoculation. Three biological replicates with three technical replicates each were performed. (**A**) Representative pictures for each day and strain (DSM25430 wt, upper row; DSM25430 Δ*itu*, lower row) are shown. Uncolonized agar is black and bacterial biomass is milky white to white. (**B**) Swarm radii were measured for each strain and time point. Data represent mean values ± SEM of three independent experiments each with three technical replicates. Statistical analysis of the difference in swarming motility between DSM25430 wt (dark gray columns) and DSM25430 Δ*itu* (off-white columns) was performed with an unpaired Student’s t-test for each time point (not significantly different: n.s., p ≥ 0.05; significantly different: *p < 0.05; **p < 0.01; ***p < 0.001; ****p < 0.0001).

We first analyzed the involvement of paenilarvin in swarming motility of *P. larvae* ERIC II. One day post inoculation, neither the wild-type nor the mutant bacteria had yet started to swarm (Fig. 6). However, after two days, the wild-type bacteria showed the expected swarming behaviour which resulted already after three days in a rather featureless mat covering the entire plate and with the original macrocolony still visible. At day six post inoculation, the inoculation site in the centre of the plate was no longer visible as area of higher cell density and instead a pattern resembling zones of consolidation or terraces (Bull’s eye^30^;) had developed (Fig. 6A, upper row). In contrast, *P. larvae* DSM25430 Δ*itu* showed delayed swarming motility: Only after three days, some swarming started at the edge of the central macrocolony and the entire plate was covered not until after six days; the central macrocolony remained clearly visible even after seven days (Fig. 6A, lower row). Measuring the swarming radii at different time points allowed a quantitative analysis of the data and confirmed statistically significant differences (day 2: p value = 0.002; day 3: p < 0.0001) in swarming motility between the wild-type and mutant bacteria (Fig. 6B). These results indicated that in the absence of the iturin-like paenilarvin swarming was significantly delayed suggesting a role of this secondary metabolite in swarming motility of *P. larvae* ERIC II. Further experiments are necessary to examine the role of paenilarvins in swarming motility in more detail.

**Figure 7:**
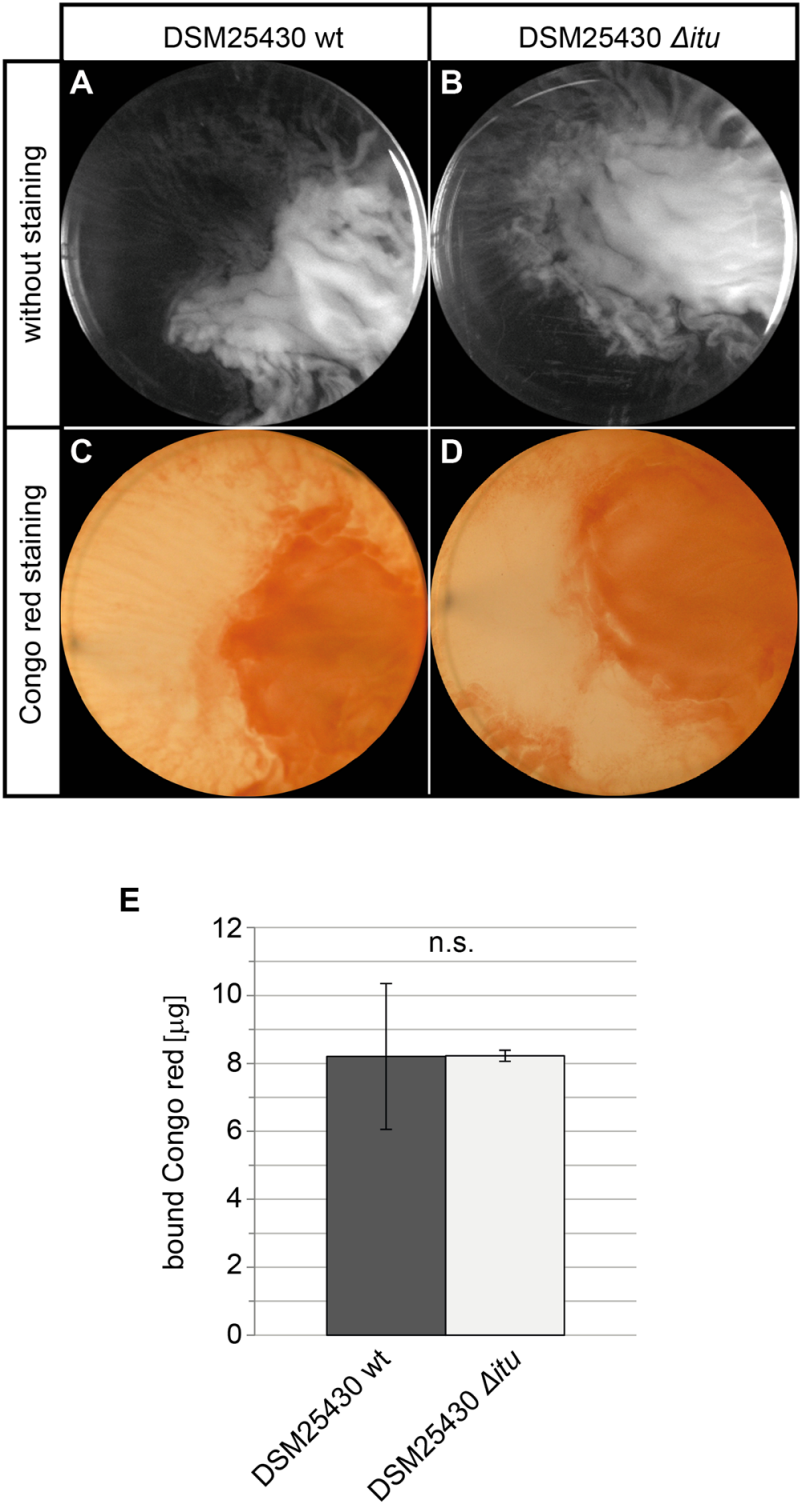
Involvement of paenilarvin in biofilm formation of *P. larvae* ERIC II. Wild-type *P. larvae* ERIC II (DSM25430 wt; **A**,**C**) and a corresponding inactivation mutant for the paenilarvin gene cluster (DSM25430 Δ*itu*; **B**,**D**) were tested in biofilm assays. Bacterial suspensions in brain heart infusion (BHI) broth were incubated without agitation in six-well-plates at 37 °C for five (**A**,**B**) and six (**C**,**D**) days. For both strains, three biological replicates were performed both for obtaining pictures under unstained conditions (**A**,**B**) and after Congo red staining (**C**,**D**). Representative pictures are shown. (**E**) Biofilm formation was quantified via determining the amount of Conge red dye retained in the biofilms upon centrifugation. Data represent mean values ± SD of three independent experiments. The difference between the wild-type and corresponding inactivation mutant was not significant (p = 0.9889, Student’s t-test).

When we tested the involvement of paenilarvin in biofilm formation of *P. larvae* ERIC II, we did not observe any difference between wild-type *P. larvae* DSM25430 and the paenilarvin gene cluster inactivation mutant *P. larvae* DSM25430 Δ*itu* (Fig. 7A–D). The biofilms formed by DSM25430 and DSM25430 Δ*itu* contained 8.21 ± 2.15 µg bound CR and 8.22 ± 0.16 µg bound CR, respectively (Fig. 7E). The difference was not significant (p = 0.9889, Student’s t-test). Therefore, biofilm formation was unaffected by the absence of paenilarvin production indicating that the iturin-like lipopeptide has no role in this process.

## Discussion

The honey bee pathogen *P. larvae* is the etiological agent of AFB, the most destructive bacterial disease of honey bees^4^. *P. larvae* is considered an obligate killer because death of the infected larva and subsequent degradation of the larval cadaver to a slimy mass that dries out to the so-called foulbrood scale consisting of *P. larvae* spores only are both necessary prerequisites for efficient spore production and transmission (for recent reviews see^61,62^). Hence, the pathogenic phase (until the death of the larva) and the saprophytic phase (degradation of the larval cadaver) are equally important phases in the *P. larvae* lifecycle wherein the death of the infected larva does not represent the end of the vegetative growth of *P. larvae* but rather marks the transition from the pathogenic to the saprophytic phase. In the recent past, the availability of the genome sequences of the *P. larvae* genotypes ERIC I and ERIC II^13^ and of protocols for the genetic manipulation of this pathogen^19,63^ has led to considerable progress in elucidating *P. larvae* virulence factors. These results improved our understanding of the bacterial strategies employed to kill the larvae, hence, strategies mainly for the pathogenic phase of the lifecycle of *P. larvae*^62^. In contrast, the saprophytic phase still remained elusive due to the lack of appropriate functional assays. We are convinced that for degrading the larval cadaver, *P. larvae* has to be capable of some kind of coordinated or cooperative multicellular behaviour like swarming motility and/or biofilm formation. Hence, the here presented swarming and biofilm assays for *P. larvae* will allow identifying factors relevant for the success of this bee pathogen after the infected larva has been killed. This will further our understanding of the entire lifecycle of *P. larvae*, which in turn is a prerequisite for the future development of sustainable treatment strategies.

Swarming motility is a coordinated bacterial activity and widespread amongst flagellated bacteria^64^. Several species of the genus *Paenibacillus* have been shown to be capable of swarming^32–34^. *P. larvae* harbors peritrichously arranged flagella, needs to rapidly colonize the larval cadaver which can be considered a nutrient-rich environment, and needs to convert larval to bacterial biomass quite fast. Hence, capability of swarming suggested itself for *P. larvae*. However, so far we could not demonstrate a general ability to swarm for the species *P. larvae*. Instead, only the *P. larvae* genotype ERIC II exhibited swarming motility in our assays. One possible reason for this difference might be the differential expression of paenilarvin, an iturin-like lipopeptide, most probably acting as biosurfactant. It has been described that in addition to flagellae and an extracellular matrix the production of biosurfactants is often required for swarming motility to occur^35^. Biosurfactants are surface active agents (hence the name) produced by microorganisms. They are amphiphilic and thus lower the surface tension or interfacial tension between either two liquids or a liquid and a solid. On agar plates, they might increase the wettability thereby facilitating spreading behaviour and swarming motility. The role of lipopeptide biosurfactants for the motility of their bacterial producers on agar plates is well characterized and it has been shown for many swarming bacteria that surface motility is lost or reduced in mutants deficient in producing lipopeptides^59,60^. In accordance with these results, the *P. larvae* ERIC II Δ*itu* mutant lacking the production of paenilarvins^29^ exhibited a significantly delayed swarming behaviour. Because this mutant does not differ in growth rate from its corresponding wild type strain^29^, growth defects cannot explain the delayed swarming phenotype. Our results rather indicate that the paenilarvins are involved in facilitating swarming motility of *P. larvae* ERIC II. Hence, we propose that the biological roles of paenilarvins comprise not only their anti-fungal activity^28^ but also their activity as biosurfactant.

It has already been demonstrated that *P. larvae* ERIC II - but not ERIC I - produces the lipopeptide paenilarvin^13,23,28^. We here showed that *P. larvae* ERIC II swarming was hampered in the absence of paenilarvin production. Hence, the inability of *P. larvae* ERIC I to swarm might be related to the lack of paenilarvin production in this genotype. However, since surface motility was only delayed but not totally lost in the paenilarvin deficient *P. larvae* ERIC II Δ*itu* mutant, paenilarvin obviously alleviated swarming but was not essential for swarming. Therefore, the lack of paenilarvin production cannot be the only explanation for the lack of swarming of *P. larvae* ERIC I. Further research is needed to elucidate the regulatory networks that control swarming in *P. larvae* and to understand the role of swarming during AFB pathogenesis and degradation of the larval cadaver.

Biofilm formation is another coordinated activity of a cooperatively acting group of bacteria. Normally, biofilms are considered sessile communities composed of one or several bacterial species embedded within an extracellular matrix and attached to a solid surface or formed as pellicles at liquid-air interfaces^35^. Recently, the additional existence of free floating biofilm aggregates has been demonstrated for *Pseudomonas aeruginosa*^40^. The ability to form biofilms had not been proposed for *P. larvae* so far, although *P. larvae*-specific FISH analysis of diseased larvae had already revealed the formation of bacterial clusters or aggregates akin to floating biofilms in the larval midgut lumen during early stages of infection^16^. Bacterially produced extracellular matrix is a hallmark and prerequisite of biofilms and, indeed, genes encoding exopolysaccharide biosynthesis proteins have been annotated in the genome of *P. larvae* (ERIC I: GenBank ETK30098.1; ERIC II: GenBank AHD06625.1)^13^. Furthermore, the slimy consistence of the bacterial mass which remains after total degradation of larval cadaver points to biofilm formation by *P. larvae* at least at the end of its lifecycle. Using biofilm specific staining methods we here demonstrated that both genotypes of *P. larvae* were able to form biofilms when incubated in broth culture without agitation, hence, in static liquid. The biofilms did neither adhere to the bottom of the wells nor did they form pellicles at the air liquid interface but instead they appeared as free floating biofilms as already described for *Pseudomonas aeruginosa*^40^. These free floating biofilms seemed to be loosely attached to the walls of the wells. For *P. larvae* degrading the larval cadaver, this cadaver does neither provide a proper “surface” to adhere to nor a liquid-air interface for the formation of a pellicle. Therefore, a free floating biofilm at the beginning of the saprophytic phase is a likely cooperative activity to ensure optimal colonization of the cadaver and access to all nutrients.

Although lipopeptides have been shown to play an important role in surface attachment and biofilm formation in *Pseudomonas* and *Bacillus*^59^, we could not demonstrate a role for paenilarvin in biofilm formation of *P. larvae*. Future research is needed to analyze *P. larvae* biofilm formation in detail. We need to unravel the steps and the regulatory mechanisms in biofilm formation, to identify the components of the extracellular matrix, and to understand the role of biofilm formation for the success of *P. larvae* as bee pathogen.

## Experimental Procedures

### Bacterial strains and culture conditions

*P. larvae* wildtype strains ATCC9545 and DSM25430 representing genotypes ERIC I and ERIC II were used in this study (Genersch *et al*.^4^). Both strains have been extensively characterized before; both are virulent and harbor all tested phenotypic features characteristic for the respective genotype^4,9–11,14,15^. In addition, the recently published mutant strain DSM25430 Δ*itu* was used which carries a paenilarvin gene cluster inactivation^29^ generated via the TargeTron Gene Knockout System (Sigma-Aldrich,Germany) following a protocol originally established for *P. alvei*^65^ and modified for use in *P. larvae*^19^. DSM25430 wt and DSM25430 Δ*itu* do neither differ in their growth characteristics nor in virulence for infected larvae^29^. Furthermore, five field strains of *P. larvae* ERIC I (00-087, 01-440, 02-130, 03-159, 03-189) and five of *P. larvae* ERIC II (00-897, 00-1163, 03-194, 03-522, 03-525), all of them isolated from contemporary AFB outbreaks, were employed in this study. These strains have already been extensively characterized and used in other studies^4,9,10,66^. Wildtype strains as well as mutant strains were cultivated on Columbia sheep blood agar plates (CSA; Oxoid, Hampshire, UK) at 37 °C for 3–5 days or according to the conditions given for each assay (see below).

### Swarming assay

Swarming assays were performed with *P. larvae* ERIC I strains (ATCC9545, 00-087, 01-440, 02-130, 03-159, 03-189) and ERIC II strains (DSM25430, DSM25430 Δ*itu*, 00-897, 00-1163, 03-194, 03-522, 03-525). To obtain pre-cultures, 2.0 ml of BHI (brain heart infusion; Merck KGaA, Darmstadt, Germany) medium were inoculated with one single colony of each *P. larvae* strain and incubated overnight at 35 °C with agitation. The next day, BHI broth (Merck, Darmstadt, Germany) agar plates (0.5%) were poured from relatively cool (~50 °C^30^;) agar. The plates were dried with the lid open at room temperature for 15 min. The pre-cultures were diluted to an OD_600_ of 0.1, and 10 µl of diluted culture were carefully pipetted into the center of each plate. The plates were then dried for another 15 min and subsequently incubated at 37 °C for several days. Pictures of the plates were taken after one, two, three, six, and seven days. The pictures were analyzed via ImageJ 1.44p to measure the radii of the areas covered by swarming *P. larvae*. Three biological replicates with three technical replicates each were performed. Data represent mean values ± SEM and statistical analysis was performed by the unpaired Student’s t-test with GraphPad Prism 6.

### Biofilm assay

Biofilm assays were performed with the *P. larvae* ERIC I strain ATCC9545 and the wildtype ERIC II strain DSM25430 and the corresponding mutant DSM25430 Δ*itu*. Pre-cultures of *P. larvae* were prepared as described above and incubated at 35 °C with agitation until the stationary phase was reached (after about 42 h). The pre-cultures in the stationary phase were diluted to an OD_600_ of 0.1 and 6.0 ml of diluted culture were transferred to the wells of a Cellstar 6-well tissue culture plate (Greiner Bio-One GmbH, Frickenhausen, Germany). Wells for negative controls contained medium only. The plates were incubated at 37 °C for five days without agitation (static culture). After five days, pictures of the unstained wells containing either cultivated (static culture) *P. larvae* or medium only (negative controls) were taken. By then, the cultures of ATCC9545 and DSM25430 had reached an OD_600_ of about 0.4 and 0.5, respectively. For better visualizing the biofilm, Congo red (CR; Merck KGaA, Darmstadt, Germany), a direct dye for staining amyloid fibres and extrapolysaccharides in biofilms^47–51^ was used. CR was added to all wells to reach a final concentration of 30 µg/ml and the plates were incubated for another 24 h at 37 °C before pictures of all wells (static culture of *P. larvae* or medium only) were taken.

To compare biofilm formation of *P. larvae* DSM25430 wildtype and the mutant strain DSM25430 Δ*itu*, binding of CR and, hence, the production of extracellular matrix was quantified. To this end, CR bound by the *P. larvae* biofilm was removed by centrifugation (5 min, 13,000 *g*) and unbound CR was determined by measuring the absorbance of the supernatant at 490 nm^67,68^. BHI medium which was incubated with CR in the same way was used as reference value for calculating the amount of bound CR^67,68^. The amounts of bound CR can be compared directly since the growth of the mutant does not differ from the wild-type^29^. Data represent mean values ± SD of three biological replicates and were analyzed by the unpaired Student’s t-test (GraphPad Prism 6).

To further substantiate that CR specifically stains the extracellular matrix and does not stain planktonic bacterial cells, the dilute pre-cultures of *P. larvae* (OD_600_ = 0.1) were incubated in the presence of CR (30 µg/ml) for 24 hours at 37 °C with constant agitation to prevent biofilm formation. Under these non-static conditions, the cultures of ATCC9545 and DSM25430 reached an OD_600_ of about 1.0 and 1.2, respectively. Subsequently, a volume of 6 ml of planktonic cells was transferred to the wells of a 6-well culture plate and pictures were taken.

To demonstrate *P. larvae* cells adherent to the walls of the culture wells, Crystal violet staining was performed by slightly modifying established protocols^69^. *P. larvae* cultures were grown overnight in BHI broth and diluted to an OD_600_ of 0.1. A volume of each 150 µl of the diluted bacterial culture was pipetted into the wells of a 96-well cell culture plate (Greiner Bio-One GmbH, Frickenhausen, Germany). Negative controls were left uninoculated. Cultures were incubated at 37 °C for six days. The plates were washed three times with MilliQ-water, stained with 0.1% Crystal violet (Merck KGaA, Darmstadt, Germany) and again washed three times before pictures were taken.

For visualizing the three-dimensional structure of the biofilms via fluorescence microscopy, thioflavin S (Sigma-Aldrich Chemie GmbH, Munich, Germany), a fluorescent dye for staining amyloid fibres and extrapolysaccharides in biofilms, was used. Pre-cultures were obtained by inoculating Sf-900 II SFM medium (Thermo Fisher Scientific, Darmstadt, Germany) with one single colony of each *P. larvae* strain followed by incubation at 37 °C with agitation overnight. The pre-cultures were diluted with SF-900 II SFM medium to an OD_600_ of 0.01 and transferred to a 96-well tissue culture plate (Greiner Bio-One GmbH, Frickenhausen, Germany). After addition of 30 µg/ml thioflavin S (Sigma-Aldrich Chemie GmbH, Munich, Germany), the plates were sealed with parafilm and incubated at 37 °C for 6 days. Thioflavin S fluorescence organized in a clearly defined 3D-structure was visualized using fluorescence inverted microscopy (Eclipse Ti, Nikon GmbH, Düsseldorf, Germany). Z-stack processing was performed to obtain the three-dimensional images. Biofilms grown in the absence of thioflavin S were included in the experiments as controls in order to determine the background fluorescence.

### Data availability

All data generated or analyzed during this study are included in this published article.
